# Editorial: Molecular Components of Store-Operated Calcium Entry in Health and Disease

**DOI:** 10.3389/fncel.2021.771138

**Published:** 2021-10-05

**Authors:** Joanna Gruszczynska-Biegala, Francisco Javier Martin-Romero, Tarik Smani, Agnese Secondo

**Affiliations:** ^1^Laboratory of Molecular Biology, Mossakowski Medical Research Institute, Polish Academy of Sciences, Warsaw, Poland; ^2^Institute of Molecular Pathology Biomarkers, University of Extremadura, Badajoz, Spain; ^3^Group of Cardiovascular Pathophysiology, Institute of Biomedicine of Seville, University Hospital of Virgen del Rocío, University of Seville, CSIC, Seville, Spain; ^4^Department of Medical Physiology and Biophysics, University of Seville, Seville, Spain; ^5^Department of Neuroscience, “Federico II” University of Naples, Naples, Italy

**Keywords:** SOCE, STIM1, STIM2, TRP channels, ORAI

Store-Operated Calcium Entry (SOCE) is a ubiquitous Ca^2+^ influx mechanism first described in 1986 (Putney, 1986). This conserved mechanism results from the interaction between the tetraspanning ORAI1 channel located in plasma membrane and the unique endoplasmic reticulum (ER) Ca^2+^ sensor, stromal interaction molecule 1 (STIM1), *via* their respective intracellular domains (Roos et al., 2005; Csutora et al., 2008). Moreover, there are two homologs of ORAI1, namely ORAI2 and ORAI3, all generating SOCE with the same mechanism.

Molecularly, upon ER Ca^2+^ depletion, STIM1 senses the filling state of ER with its N-terminus characterized by a low affinity for Ca^2+^ ion (200 nM-600 microM). After dimerization, STIM1 diffuses to the plasma membrane regions where it interacts with ORAI1 inducing SOCE through Calcium Release-Activated Calcium (CRAC) channel opening. This event may mediate a localized increase in the intracellular Ca^2+^ concentration useful to recharge ER of Ca^2+^ content by sarco/endoplasmic reticulum Ca^2+^-ATPase (SERCA) intervention. Besides STIM1, also STIM2 may induce activation of ORAI1 (Brandman et al., 2007) but with a weak Ca^2+^ entry that is nevertheless able to trigger NFAT1 activation (Son et al., 2020).

Accumulated evidence suggests that SOCE dysfunction may produce Ca^2+^ dyshomeostasis in both excitable and non-excitable cells, thus participating to the pathogenesis of a large spectrum of diseases most of which are due to the modification of ORAI1/STIM1 interaction in consequence of changes in their expression or following a disruption of SOCE machinery. However, genetic modification of the two major players could also occur. For instance, loss of function mutations of ORAI1 and STIM1/STIM2 abolishes SOCE, thus causing autoimmunity and severe combined immunodeficiency (SCID)-like diseases. In contrast, autosomal dominant gain-of–function mutations in ORAI1 and STIM1 determine a sustained increase in CRAC and SOCE causing a large spectrum of diseases like Stormorken syndrome and nonsyndromic tubular aggregate myopathy (TAM) (Lacruz and Feske, 2015). In this respect the study by Conte et al. shows some of the adaptive or compensatory mechanisms able to counteract the genetic-encoded Ca^2+^ dyshomeostasis and describes the alteration in the differentiation process of muscle cells deriving from TAM-patients carrying STIM1 L96V mutation.

Mechanistically, mutations in the transmembrane domains of the pore region of ORAI channels may produce SOCE alterations. Of note, the elucidation of the isoform-specific mutations may provide useful targets for selective drug development. In this context, the study by Tiffner et al. shows the molecular consequences of the enhanced hydrophobicity along TM3 of ORAI1 or ORAI3 structure on the gain-of-function of SOCE and CRAC currents.

In this Research Topic, two manuscripts review the role of ORAI1 in cardiovascular remodeling and heart failure. The first article by Luo et al. provides an overview of the new role of ORAI1-mediated SOCE in the maladaptive cardiac hypertrophy and heart failure. The review discusses the current knowledge regarding the involvement of ORAI1 in hypertrophic development induced by neurohormonal stimulation *in vitro* or *in vivo*, using transgenic mice subjected to different procedures that induce cardiac hypertrophy and consequently heart failure.

The other review article by Shawer et al. provides a broad perspective of the role ORAI1-mediated vascular smooth muscle cell (VSMC) switching from contractile to synthetic phenotypes, a critical step for their proliferation and migration. Authors focus on the involvement of ORAI1 in the pathological vascular remodeling related to atherosclerosis, neointimal hyperplasia and restenosis. They also discuss the potential mode of action of a large list of SOCE inhibitors examined in different cell lines.

Both reviews consider that new pharmacological selective inhibitors are valuable tools to study the role of SOCE in health and disease, which might pave the way for the development of therapeutic ORAI1 inhibitors to mitigate pathologic cardiac and vascular remodeling.

Galeano-Otero et al. in an original article related to the circulatory system highlight for the first time the role of SARAF, SOCE-associated regulatory factor, in the activation of endothelial cells and angiogenesis. The study nicely demonstrates that SOCE participates in several steps of angiogenesis, such as endothelial cell proliferation and migration, tube formation, and sprouting, and shows that SARAF co-localizes and interacts with ORAI1, which might sustain an enhanced Ca^2+^ entry in these highly proliferative cells during angiogenesis.

Since the discovery of the molecular components of SOCE, including the STIM, ORAI and TRP proteins, a growing number of publications describe their functions in the healthy and diseased central nervous system (CNS) (Serwach and Gruszczynska-Biegala, 2019). In this regard, Zhang and Hu summarize the current literature reporting on the variable expression of STIM, ORAI and TRPC in neurons and glial cells from different parts of the CNS. The authors also provide a systematic overview describing the current understanding of the physiological and pathological role of SOCE and its molecular components in each of these brain regions. The impact of existing therapies on SOCE is also reviewed.

Two additional review articles describe the relationship between Huntington's disease (HD) and SOCE (Latoszek and Czeredys). As an inherited neurodegenerative disorder, HD is characterized by the loss of γ-aminobutyric acid (GABA)-ergic medium spiny neurons (MSNs) in the striatum (Vonsattel and DiFiglia, 1998), and SOCE has been shown to be elevated in several HD models. Czeredys's review provides a comprehensive update on the implications of SOCE, STIM2, ORAIs and TRPCs in HD pathology using various HD models, including YAC128 mice (HD transgenic model), HD cellular models, and induced pluripotent stem cell-based GABAergic MSNs that are obtained from fibroblasts of adult HD patients. The author further discusses potential drug candidates that may restore normal SOCE and consequently prevent dendritic spine loss in HD. Latoszek and Czeredys review recent data indicating that HD is also a neurodevelopmental disease, as neuronal cells differentiated from juvenile HD patient-derived iPSCs show deficits in development and adult neurogenesis. Finally, the authors also review different protocols to obtain MSNs and brain organoids as powerful tools to study HD.

Also related to HD, Vigont et al. in an original article, demonstrate the role of STIM2 in the elevated Ca^2+^ entry in HD cellular models. More specifically, these authors generated MSNs modeling a juvenile form of HD and show high levels of SOCE using patch-clamp. Upregulation of STIM2 protein expression was also observed, and the shRNA-mediated suppression of STIM2 expression attenuated SOCE. For this reason, Vigont et al. used the anti-HD drug EVP4593 to demonstrate that this drug decreased huntingtin expression and also the expression of STIM2, postulating that STIM2 could be a novel therapeutic target for HD.

In addition to the main components of SOCE, being the STIM and ORAI proteins, an increasing number of proteins have been also reported to play an important role in the STIM-ORAI dependent regulation of SOCE. Serwach and Gruszczynska-Biegala thoroughly review and summarize the current knowledge about STIM protein target molecules, including positive (mGluR, septins, synaptopodin, POST, EB and Golli proteins) and negative (Homer, SARAF, presenilin1, and NEUROD2) regulators and effectors [L-type voltage-operated Ca^2+^ channels (VOCCs) and receptors such as AMPAR and NMDAR] in the CNS. This review also highlights the importance of the interaction of STIM proteins with their target proteins in pathology, such as hypoxic/ischemic neuronal injury, epilepsy, Alzheimer's, Huntington's and Parkinson's diseases, and in physiological conditions of the CNS.

Finally, in this issue Coronas et al. review the role of Ca^2+^ channels in neural stem cells (NSCs) and glioblastoma stem cells (GSCs), derived from oncogenic mutations in adult NSCs and responsible for the emergence of malignant brain tumors. The review focuses on the Ca^2+^ toolkit and its physiological role in GSCs, in NSCs and their progenies to show that stemness is controlled by VOCCs, store-operated Ca^2+^ channels (SOCs), IP_3_Rs in NSCs, and by VOCCs, nicotinic receptors and TRPVs in GSCs.

In summary, this special issue of Frontiers in Cellular Neuroscience provides a comprehensive overview of the most recent data on the role of SOCE and related proteins: STIMs, ORAIs and others that regulate them, in the physiology and pathology of a variety of cells. It highlights the importance of SOCE and its regulatory proteins as potential drug targets for future therapies against neurodegenerative, cardiovascular or muscular diseases and brain tumors.

## Author Contributions

All authors listed have made a substantial, direct and intellectual contribution to the work, and approved it for publication.

## Funding

JG-B was supported by the National Science Centre grant (research project no. 2017/26/E/NZ3/01144), FJM-R was supported by the Spanish Ministry of Science and Innovation (research project BFU2017-82716-P), TS was supported by the Spanish Ministry of Science and Innovation (research project no. PID2019-104084GB-C22/ AEI/10.13039/501100011033), AS was supported by PRIN2015–Prot. 2015KRYSJN (Italian Ministry of Education and Research), Progetto Speciale di Ateneo CA.04_CDA_n_103 27.03.2019 and Progetto di Ateneo Linea A CdA_54_2020_FRA.

## Conflict of Interest

The authors declare that the research was conducted in the absence of any commercial or financial relationships that could be construed as a potential conflict of interest.

## Publisher's Note

All claims expressed in this article are solely those of the authors and do not necessarily represent those of their affiliated organizations, or those of the publisher, the editors and the reviewers. Any product that may be evaluated in this article, or claim that may be made by its manufacturer, is not guaranteed or endorsed by the publisher.
